# Cyanobacterial Allelochemicals But Not Cyanobacterial Cells Markedly Reduce Microbial Community Diversity

**DOI:** 10.3389/fmicb.2017.01495

**Published:** 2017-08-08

**Authors:** Filipa Dias, Jorge T. Antunes, Tiago Ribeiro, Joana Azevedo, Vitor Vasconcelos, Pedro N. Leão

**Affiliations:** ^1^Interdisciplinary Centre of Marine and Environmental Research (CIIMAR/CIMAR), University of Porto Matosinhos, Portugal; ^2^Department of Biology, Faculty of Sciences, University of Porto Porto, Portugal

**Keywords:** allelopathy, cyanobacteria, LEGE 05292, portoamides, *Phormidium*

## Abstract

The freshwater cyanobacterium *Phormidium* sp. LEGE 05292 produces allelochemicals, including the cyclic depsipeptides portoamides, that influence the growth of heterotrophic bacteria, cyanobacteria, and eukaryotic algae. Using 16S rRNA gene amplicon metagenomics, we show here that, under laboratory conditions, the mixture of metabolites exuded by *Phormidium* sp. LEGE 05292 markedly reduces the diversity of a natural planktonic microbial community. Exposure of the same community to the portoamides alone resulted in a similar outcome. In both cases, after 16 days, alpha-diversity estimates for the allelochemical-exposed communities were less than half of those for the control communities. Photosynthetic organisms, but also different heterotrophic-bacteria taxa were found to be negatively impacted by the allelochemicals. Intriguingly, when *Phormidium* sp. LEGE 05292 was co-cultured with the microbial community, the latter remained stable and closer to non-exposed than to allelochemical-exposed communities. Overall, our observations indicate that although under optimal growth conditions *Phormidium* sp. LEGE 05292 is able to synthesize potent allelochemicals that severely impact different microorganisms, its allelopathic effect is not pronounced when in contact with a complex microbial community. Therefore, under ecologically relevant conditions, the allelopathic behavior of this cyanobacterium may be regulated by nutrient availability or by interactions with the surrounding microbiota.

## Introduction

The shaping and dynamics of aquatic microbial communities is dependent on several abiotic (e.g., nutrient levels, salinity, light quality and quantity, mixing) and biotic (e.g., grazing, competition, cell–cell contact, viruses) factors (Fuhrman, 2009; Yu et al., 2014). One particularly elusive phenomenon that is likely to influence the growth and physiological status of planktonic organisms is allelopathy, the active release of compounds – allelochemicals – that exert a direct or indirect effect on the surrounding biota, influencing their growth, physiology or behavior (Leflaive and Ten-Hage, 2007; Leão et al., 2009b). The study of allelopathic interactions in aquatic ecosystems has been mostly carried out under simplistic experimental set-ups. In particular, two types of studies (physically separated co-cultures and/or filtrate addition) using typically one producing (allelopathic) and one target (sensitive) organism have contributed to the bulk of the literature on aquatic allelopathy (Legrand et al., 2003; Leão et al., 2009b). These experimental approaches allow control over other ecological phenomena such as cell–cell interactions or nutrient competition. Extrapolation of laboratory-detected allelopathic behavior to a role in the natural setting is, however, experimentally challenging (Legrand et al., 2003; Inderjit, 2006) due to the numerous and complex network of biotic and abiotic interactions occurring in diverse microbial communities (e.g., Jonsson et al., 2009). Until recently, our ability to follow microbial community composition in detail was technically limited (Birtel et al., 2015), hindering experimental approaches for the study of allelopathy at the microbial community-level. Still, even early studies on allelopathy in lake microbial communities hinted at an important role for allelochemicals in shaping future community dynamics (Keating, 1977, 1978). The advent of affordable, high-throughput sequencing technologies has created an opportunity for experimentally interrogating chemically mediated biotic interactions in complex communities. For example, these technologies have enabled investigations of the role of natural small-molecules in the gut microbiota (e.g., Thompson et al., 2015).

Cyanobacteria, ubiquitous in the photic zone of aquatic ecosystems, are among the organisms capable of exhibiting allelopathic properties. For example, their dominance following explosive growth in certain freshwater ecosystems has been attributed in part to an ability to release allelochemicals to the surrounding medium (Leão et al., 2009b). Still, and although allelopathic behavior has been well-documented for many cyanobacteria, we currently know but a handful of cyanobacterial allelochemicals (Leão et al., 2012a). This is the case of the portoamides A and B, cyclic dodecapeptides that are likely produced by a hybrid polyketide synthase/non-ribosomal peptide synthetase pathway, together with the apparently less potent undecapepetides portoamides C and D (Leão et al., 2010). The portoamides are actively released to the culture medium by the cyanobacterium *Phormidium* sp. LEGE 05292 (formerly *Oscillatoria* sp. LEGE 05929, hereafter referred to as PHO) (Leão et al., 2010). Exposure studies with laboratory cultures of different phytoplanktonic organisms demonstrated that portoamides A and B inhibit the growth of eukaryotic microalgae *Chlorella vulgaris* and *Ankistrodesmus falcatus* as well as that of the cyanobacterium *Cylindrospermopsis raciborskii* (Leão et al., 2010). The growth of several gram-positive and gram-negative heterotrophic bacteria was inhibited by the lyngbyazothrins (Zainuddin et al., 2009), compounds that are likely identical to the portoamides but isolated from another cyanobacterium. In addition, the spent media of PHO cultures was also deleterious to the growth of different eukaryotic microalgae (Leão et al., 2009a, 2010). Because PHO allelochemicals affect diverse organisms *in vitro*, it is pertinent to study the extent to which PHO allelochemicals can impact natural microbial communities. With this in mind, in a previous study we have exposed, under laboratory conditions, a *Microcystis*-dominated microbial community from pond water to the allelochemicals exuded by an PHO culture (Leão et al., 2012b). Using microscopy counts and DGGE analysis, we showed that the allelochemicals caused both positive and negative growth effects on different phyto- and zoo-planktonic organisms, but mostly impacted negatively the growth of eukaryotic microalgae and cyanobacteria. Moreover, we observed that different *Microcystis* genotypes had distinct responses to exposure to PHO allelochemicals – supporting the notion that chemically mediated interactions are a driving force for intraspecific diversification in phytoplankton [as observed for example for dinoflagellates (John et al., 2015)].

Here, we aimed to deepen our understanding on how microbial communities may be influenced by the presence of an allelopathic organism or its allelochemicals, by exploiting the fingerprinting potential of massive parallel sequencing technology. We harvested a planktonic microbial community from the surface of an urban pond and acclimated it to laboratory conditions. This community was exposed to three parallel treatments: (i) an organic extract from the spent medium from a 15-day-old PHO culture, (ii) a mixture of the allelochemicals portoamides A and B at a concentration of 1 μg mL^-1^, and (iii) a control, organic extract of the medium used to culture PHO. We have also performed an additional treatment whereby PHO was inoculated at 1 × 10^4^ cells mL^-1^ in flasks containing the microbial community. Using 16S rRNA gene amplicon massive parallel sequencing, we analyzed the composition of the treated communities immediately after exposure and at two later time points (after 6 and 16 days). We found that exposure to spent medium or portoamides led to a marked decrease in community richness, while only a few (potentially opportunistic) groups were able to increase their relative abundance. Interestingly, PHO cell presence did not lead to a decrease in diversity of the community, which remained stable and comparable to the control situation.

## Materials and Methods

### Analytical Instrumentation and Procedures

^1^H NMR data were acquired on a 400 MHz Bruker Avance III spectrometer. LC-HRESIMS data for the spent medium extract and the purified portoamide mixture were acquired on an Accela HPLC fitted with a Gemini C18 column (5 μm, 110 A, 4.6 mm ID × 150 mm, Phenomenex) column, coupled to an Accela PDA detector, Accela autosampler, and Accela 600 pump and to an LTQ Orbitrap XL spectrometer, controlled by LTQ Tune Plus 2.5.5 and Xcalibur 2.1 (Thermo Scientific). Twenty microliters of each sample were injected at a concentration of 0.1 mg mL^-1^ (MeOH). The separation was carried out using a gradient from 20% MeCN (aq) to 100% MeCN over 30 min. The LTQ spectrometer was operated in positive ion mode, the capillary voltage of the electrospray ionization source (ESI) was set to 3.0 kV and the capillary temperature was 300°C. For the separation and analysis leading to the isolation of portoamides A and B, an HPLC system composed of an Alliance 2695 HPLC (Waters) coupled to a PDA 2998 detector, fitted with a XB-C18 Aeris PEPTIDE column (150 mm × 4.6 mm, 3.6 μm, Phenomenex, kept at 35°C during the chromatography) was used. Monitored wavelengths during separation were 210 and 280 nm.

Solvents used were MS-grade or HPLC-gradient grade for MS and HPLC procedures, and ACS grade for extraction and column chromatography. NMR solvents were acquired from BDH Prolabo (VWR).

### Culture Conditions

Stock cultures of the cyanobacterium PHO – formerly *Oscillatoria* sp. LEGE 05292 (Leão et al., 2009a, 2010) – were maintained in Z8 medium (Kotai, 1972), at 25°C and under a 14:10 h light (∼30 μmol photons m^-2^ s^-1^)/dark cycle. This unicyanobacterial culture has not been determined to be axenic, but no contamination is visible in exponentially growing cultures by light microscopy under high magnification, probably due to the antibiotic properties that have been attributed to the portoamides.

### Purification of Portoamides A and B

**FIGURE 1:**
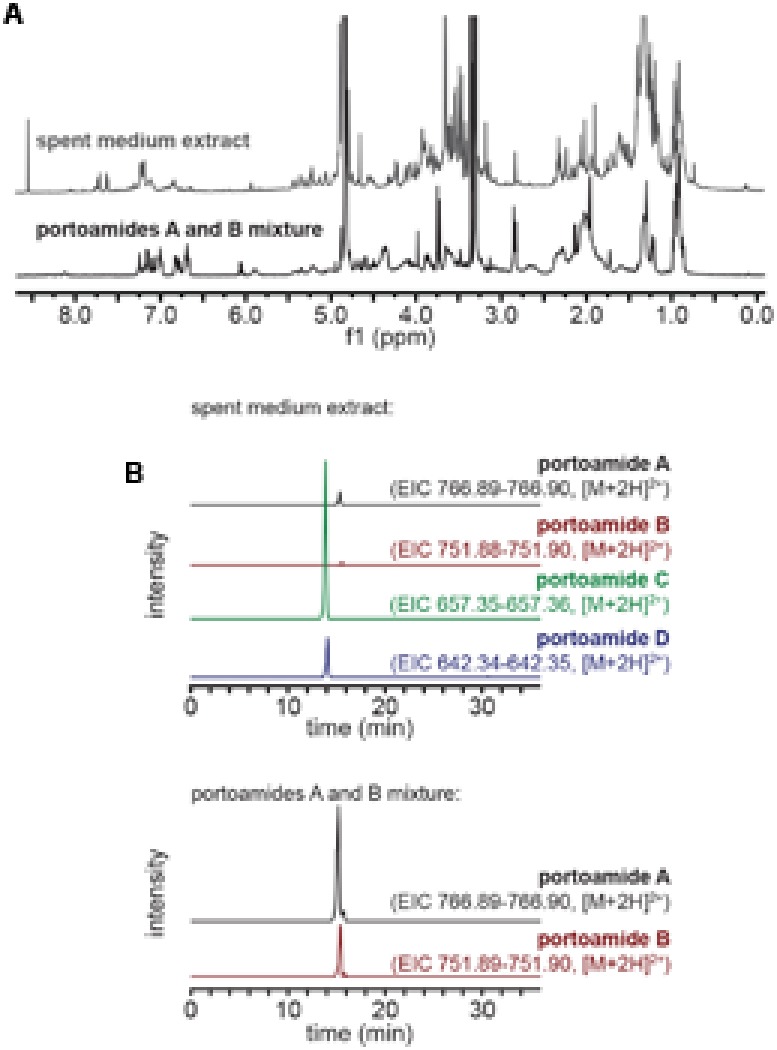
Characterization of the spent medium extract and portoamide mixture samples. **(A)** ^1^H NMR (CD_3_OD, 400 MHz) profiles of the spent medium extract and purified portoamides A and B mixture. **(B)** Extracted ion chromatograms (EICs) for portoamides obtained from LC-HRESIMS analyses of the spent medium extract (top) and portoamides A and B mixture (bottom) – the intensity scale is the same for EICs obtained from the same sample.

To obtain a mixture of portoamides A and B in the proportions found inside PHO cells, we carried out a slightly modified version of a previously reported isolation (Leão et al., 2010). Briefly, biomass from exponentially growing cultures of PHO was harvested by centrifugation, lyophilized and repeatedly extracted with a warm (<40°C) mixture of CH_2_Cl_2_/MeOH (2:1). The vacuum liquid chromatography (VLC) fractionation of this crude extract was performed on a normal phase (Si gel 60, 0.015–0.040 mm, Merck KGaA) column with an elution gradient of increasing polarity, from 3:2 EtOAc/*n*-hexane to EtOAc to MeOH. The fraction eluting with 100% MeOH contained the portoamides (from HPLC analysis) and was further fractionated by analytical-scale HPLC. A gradient from 50% MeOH (aq.) to 100% MeOH (1 mL min^-1^) was used for both the analysis and isolation of the portoamides A and B, whose mixture eluted and was collected between t_R_ = 13.0–15.0 min over multiple runs. The purity of the portoamide mixture was confirmed by ^1^H NMR, namely by integration and comparison with previously reported portoamides spectral data (Leão et al., 2010); the proportion of the mixture was found to be 2.7:1 on the basis of integration of LC-HRESIMS peaks for each compound (**Figure 1**).

### Spent Medium and Fresh Medium Extract Preparation

The spend medium (1.5 L) of a 15-day-old culture of PHO in Z8 medium, grown in the same conditions as batch cultures, was collected by filtration (0.2 μm). The filtrate was extracted using an octadecyl (C18) silica solid-phase extraction (SPE) cartridge (50 g, Strata, Phenomenex). After loading the sample onto the pre-conditioned cartridge, the column was washed first with 360 mL H_2_O, then with 2% MeOH (aq), before eluting in a single step with 720 mL MeOH. The solvent in the eluate was removed in vacuo to yield 17.8 mg of spent medium extract. The same extraction procedure was applied to fresh Z8 medium to serve as a control. The composition of the spent medium extract was assessed by LC-HRESIMS and ^1^H NMR (**Figure 1**).

### Microbial Community Harvest and Acclimation

A surface water sample from an urban park pond in Porto, Portugal (41°10′4.27′′ N, 8°40′26.06′′ W), was collected on May 27, 2016, immediately brought to the laboratory and filtered through 40 μm and then 20 μm plankton nets, to obtain homogeneous and reproducible subsamples for the exposure experiment. The filtered sample containing the <20 μm microbial community was acclimated to laboratory conditions by being placed under the light and temperature conditions described above for batch cultures, for a period of 48 h, and was then used for the exposure experiment.

### Exposure Experiment

A simplified scheme of the experimental design for the exposure experiment is provided (Supplementary Figure S1). The laboratory-acclimated microbial community was separated into four 500 mL subsamples. Each of these subsamples was used to prepare one of four different treatments: (i) fresh medium extract (control), (ii) spent medium extract, (iii) portoamides A and B, and (iv) co-culture with PHO. To prepare the fresh medium extract, one third of the obtained extract (corresponding to 500 mL of fresh medium) was transferred to a round bottom flask using MeOH and the solvent removed in vacuo before adding the 500 mL microbial community subsample to dissolve the extract residue. The same procedure was carried out for the spent medium extract. For the portoamides A and B treatment preparation, 0.5 mg of the previously obtained portoamides A and B mixture (2.7:1, respectively) was transferred to a round-bottom flask using MeOH, the solvent was then removed under reduced pressure. A 500 mL subsample of the microbial community was added to the flask so as to dissolve the portoamide residue. Finally, to prepare the PHO cell co-culture treatment, a 2.3 mL aliquot from a concentrated 15-day-old PHO culture cell suspension in fresh Z8 medium was added to a 500 mL microbial community subsample to yield 1 × 10^4^ PHO cells mL^-1^ (estimated by OD at 750 nm, using a previously obtained linear calibration curve between OD and cell density from hemocytometer count). To adjust for this step, the same volume (2.3 mL) of fresh Z8 medium was added to the round bottom flasks containing each of the other three treatments. After the different treatments were prepared, 450 mL of each treatment were distributed equally by nine 25 cm^2^ vented (0.2 μm) tissue culture flasks (50 mL per flask). Three flasks from each treatment were retrieved immediately for DNA extraction (day 0) while the remaining flasks were placed under the light and temperature conditions described above for batch cultures. Flasks were vigorously shaken twice daily; three flasks from each treatment were retrieved after 6 and 16 days of exposure and used for DNA extraction.

### DNA Extraction

Each flask retrieved from the exposure experiment was vigorously shaken and its contents transferred to a 50 mL falcon tube before centrifugation at 4500 × *g* for 10 min. The pellet was resuspended in a minimum volume of fresh Z8 medium, transferred to a 2 mL microcentrifuge tube and centrifuged at 7000 × *g* for 5 min. The supernatant was carefully removed and the pellet used for eDNA extraction which was carried out with a commercial kit (PowerSoil DNA isolation kit, MoBiO), according to the manufacturer’s instructions. For two samples corresponding to day 0 (one from the co-culture with PHO cells and one for the portoamides exposure) we were unable to obtain eDNA – given the high homogeneity of samples at day 0, this had a negligible impact on our analysis.

### DNA Sequencing and Sequence Analysis

The eDNA samples were used for paired-end massive parallel sequencing of the V3 region of the 16S rRNA gene (341F-785R primers, Klindworth et al., 2013) using the illumina MiSeq platform, which was carried out elsewhere (LGC Genomics). Obtained reads were demultiplexed using bcl2fastq 1.8.4 software (Illumina), sorted and adapter clipped. Those reads with a final length of less than 100 bases were discarded and the remaining reads were primer clipped. Forward and reverse reads were combined using BBMerge 34.48^1^ to yield a total of 866,328 reads distributed among 34 samples (average 25,480; minimum 7,317; maximum 46,654 reads). An additional pre-processing step was carried out for quality filtering in QIIME (Caporaso et al., 2010), using a maximum unacceptable Phred quality score of Q 20. A QIIME pipeline was carried out to remove chimeric sequences. After quality filtering and chimera removal, a total of 397,546 sequences were obtained across all samples (average 11,692; minimum 3,485; maximum 24,524). OTUs (closed reference) were picked in QIIME using usearch v6.1 (Edgar, 2010) and the Greengenes (McDonald et al., 2012) reference (with clustering at 97% sequence identity) and taxonomy datasets (v13.8). Before analysis, the dataset was rarefied using the smallest sample depth (3,485 sequences). Abundance and diversity analyses were carried out using the Core Diversity Analyses workflow in QIIME and with the phyloseq package for R (McMurdie and Holmes, 2013). Beta diversity was estimated by weighted UniFrac pairwise distance analysis. Abundance differences between treatments for each taxon and for a particular day were evaluated statistically using one-way ANOVA, and for taxa with significant (*P* < 0.05) differences, Fisher’s unprotected LSD test was carried out to identify significant (*P* < 0.05) differences between treatments for the same day. Sequencing reads for this study were deposited in the European Nucleotide Archive under accession number PRJEB21598.

## Results and Discussion

### Characterization of the Spent Medium Extract

The extract containing organic components from the spent medium of a 15-days-old PHO culture, obtained by passage of the medium through a reversed phase SPE column, was analyzed by ^1^H NMR and compared to the ^1^H NMR spectra of the portoamides A and B mixture (**Figure 1A**). Comparison of the two spectra indicated that portoamides A and B were not main components of the extract (as clearly perceived from the δ8.0-5.5 region of both spectra). To clarify whether the portoamides were in fact present in the spent medium extract, LC-HRESIMS was carried out and portoamides A, B, C, and D were detected with a proportion of (12:1:62:4, **Figure 1B**).

### Initial Community Composition

**FIGURE 2:**
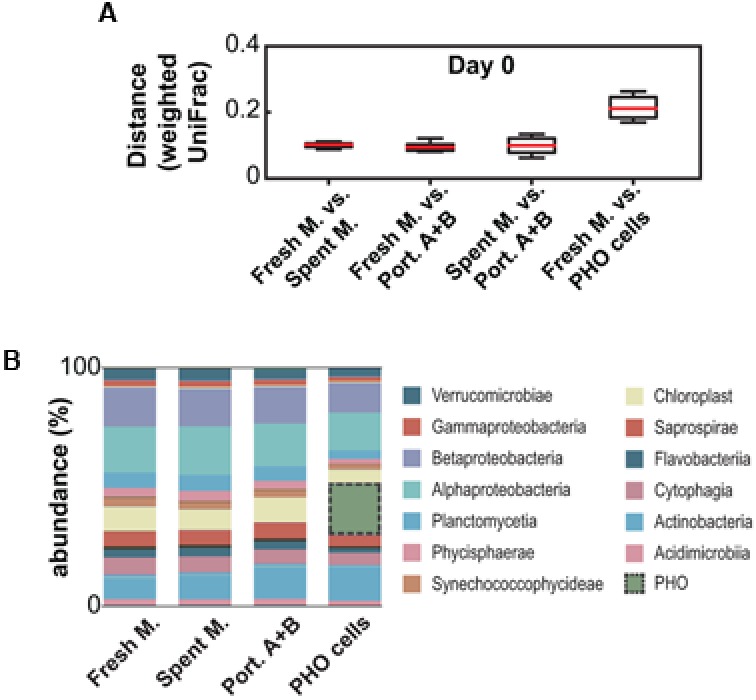
Analysis of the initial community compositions (day 0) from 16S rDNA amplicon metagenomics data. **(A)** Weighted UniFrac-based pairwise distance comparison between different treatments. **(B)** Community composition (relative abundances, averaged) at the class/subclass level.

Immediately after exposure, as expected, the microbial community was comparable among all treatments with the obvious exception of the treatment in which PHO was inoculated at 1 × 10^4^ cells mL^-1^ (**Figure 2A**). This established that the subsampling was effective and that the communities were equivalent among samples at the beginning of the experiment (day 0). The initial communities were found to be mainly composed (at the class/subclass level) of Alphaproteobacteria, Betaproteobacteria, Actinobacteria and chloroplast lineages (**Figure 2B**). However, due to the plankton net filtration step, this may not entirely reflect the composition of the original community present in the urban pond.

### Exposure to PHO Allelochemicals Drastically Reduces Diversity

**FIGURE 3:**
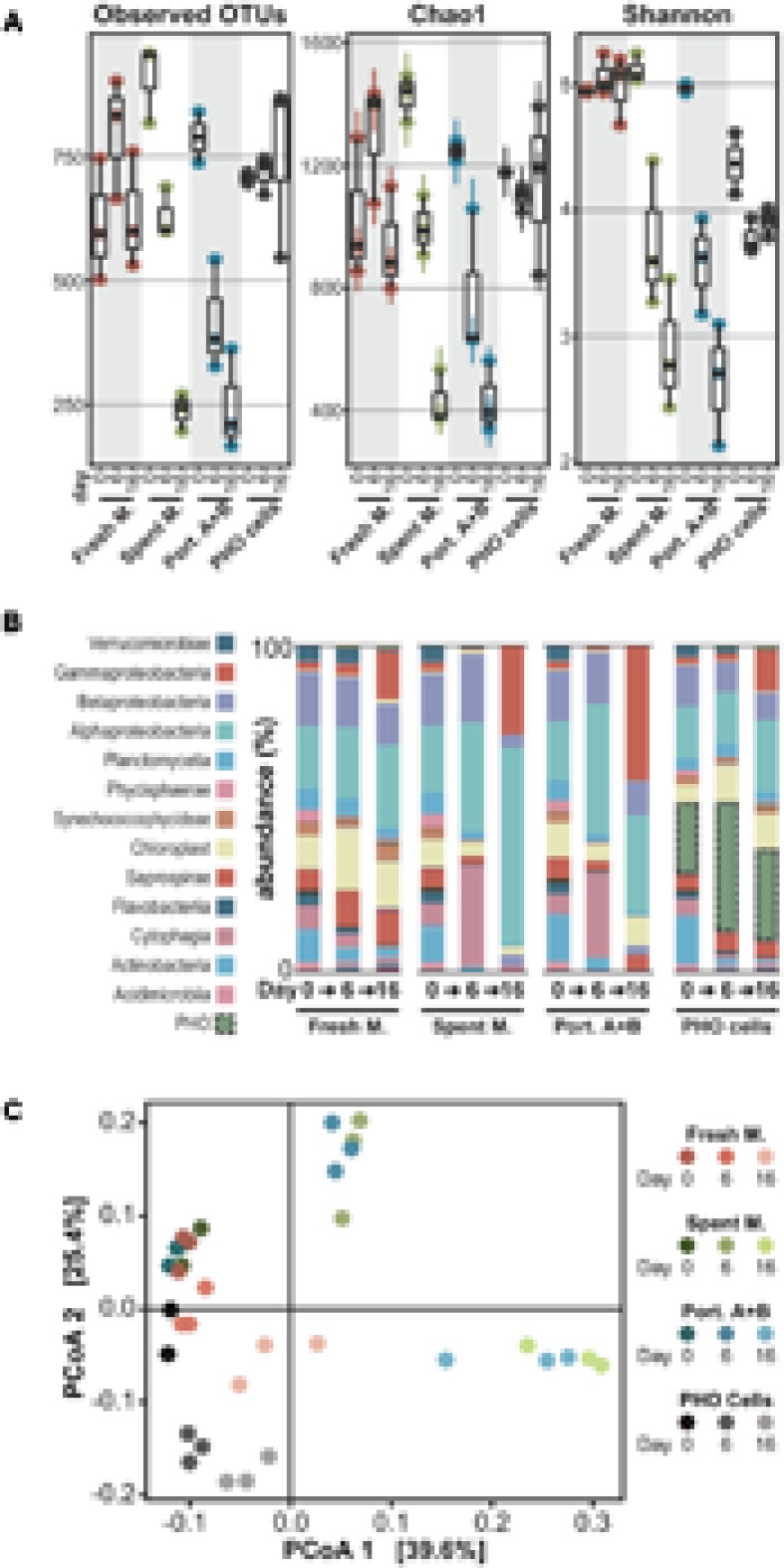
Analysis of 16S rDNA amplicon metagenomics data for the microbial communities at different time points following exposure. **(A)** Box-whisker plots depicting alpha diversity indexes for each of the microbial communities analyzed; **(B)** Community compositions (relative abundances, averaged) at the class/subclass level; **(C)** Principal coordinates analysis visualization of the weighted UniFrac distances between the microbial communities.

Comparison of the microbial community compositions after 6 and 16 days of exposure revealed that profound changes occurred in communities exposed to the organic extract from PHO spent medium and to the 1 μg mL^-1^ mixture of portoamides A and B – i.e., the lower limit for the interval of estimated concentrations of the compounds in PHO culture filtrates (Leão et al., 2010) – when compared to the control communities that were exposed to a fresh medium organic extract (**Figure 3**). In the allelochemical-exposed treatments, alpha diversity – as estimated by the chao1 index – decreased (30%, spent medium; 38% portoamides) after 6 days of exposure and decreased even further by the end of the experiment (70%, spent medium; 65%, portoamides) while for the fresh medium extract, alpha diversity increased slightly after 6 days and by the end of the experiment was comparable to the day 0 communities (**Figure 3A**). Beta diversity analysis indicated that after 6 and 16 days of exposure, the distance between allelochemical-exposed treatments was consistently lower than the distances between each of these and the control treatment (**Figure 3C**). This suggests that the allelopathic effect of either the portoamides A and B alone or that of the spent medium components drives the community in the same direction, which is not surprising given that portoamides are found in the spent medium extract (**Figure 1B**).

It is possible that our analysis missed rare taxa and that the observed decrease in evenness (**Figure 3A** and Supplementary Figure S2) also led to an underestimation of species richness. Still, loss of diversity can be expected when exposing microbial communities to compounds with antibiotic properties, as several studies on the effects of commercial antibiotics on gut microbiota have demonstrated (Dethlefsen and Relman, 2011; Zaura et al., 2015). In fact, when a soil community was exposed to weekly pulses of the natural antimicrobial macrolactins (∼2.5 μg/g soil) a 16% loss in alpha diversity (chao1) was observed over 4 weeks (Yuan et al., 2016) – a considerably milder impact than the one herein reported over a shorter exposure period.

### Effect of PHO Allelochemicals on Specific Taxa

**FIGURE 4:**
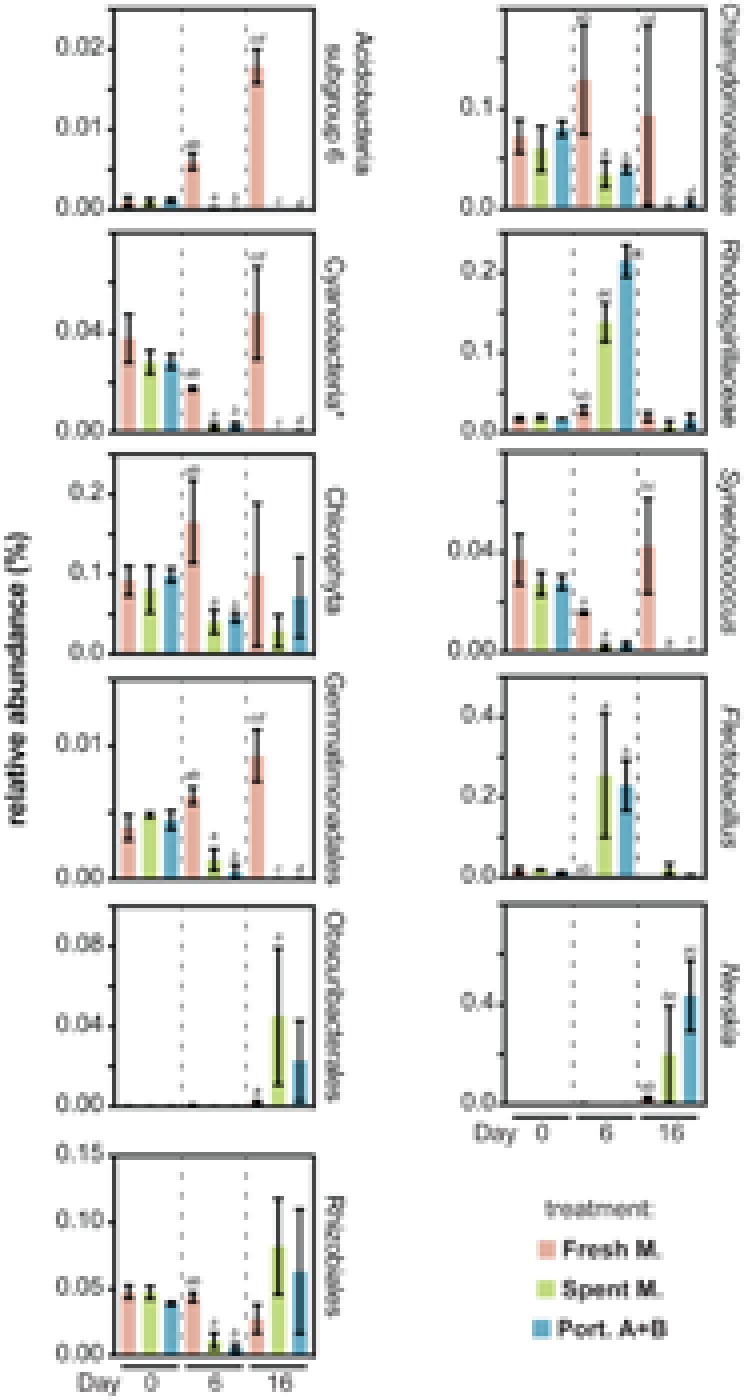
Relative abundances of specific taxa found to be differentially impacted by the fresh medium, spent medium and/or portoamides treatments. For a given chart, pairs of bars labeled with the same letter denote statistically significant (95% confidence level) differences for the respective values. ^∗^Excluding chloroplasts.

We observed that, at different taxonomic levels, several taxa were strongly affected by exposure to the allelochemicals (**Figure 4**). Most of these decreased their abundance in the presence of allelochemicals, while a few had abundance peaks at days 6 or 16. Among those taxa negatively affected by allelochemical treatments are eukaryotic (Chlorophyta in general, but particularly Chlamydomonadaceae) and prokaryotic (Cyanobacteria, most prominently *Synechococcus* spp.) photoautotrophs that can potentially compete for light and nutrients with PHO, in line with our previous observations (Leão et al., 2009a, 2010, 2012b). In particular, our previous study on the exposure of a *Microcystis* spp.-dominated planktonic community to PHO allelochemicals (Leão et al., 2012b) revealed mostly inhibitory effects on cyanobacteria and eukaryotic microalgae, although some cyanobacteria (including specific *Microcystis* genotypes) were positively impacted by exposure. Other taxa that were negatively impacted include the Acidobacteria subgroup 6, which steadily increased in abundance in the absence of allelochemicals but was practically absent in allelochemical-exposed treatments, similar to what was observed for the Gemmatimonadales. Under allelochemical exposure, the Rhizobiales had a decreased abundance when compared to the control only at the “day 6” time point. Conversely, at the same time point, Rhodospirillaceae and members of the genus *Flectobacillus* exposed to allelochemicals were found at considerable higher levels than in the fresh medium control. These transient abundance peaks could be related to the generation or disappearance of specific niches – for example due to the presence of the allelochemicals which can potentially be degraded by certain microorganisms [many genera within the family Rhodospirillaceae are known to be chemoorganotrophs (Garrity et al., 2015)]. Yet another abundance pattern observed during this experiment was displayed by the cyanobacteria-related Obscuribacterales (Soo et al., 2014), as well as by the neustonic *Nevskia* spp. – these taxa showed a sharp increase in abundance by the end of the experiment in the allelochemical-treated conditions, having exhibited very low abundances in all communities at both days 0 and 6. Due to the experimental setup (batch cultures) used in our study, it is unclear if these transient dynamics elicited by the allelochemical disturbance would lead to an alternative stable state (Scheffer et al., 1993; Shade et al., 2012) or if the community was resilient enough to revert back to its original state. Nevertheless, decreased OTU richness and evenness (**Figure 3A** and Supplementary Figure S2) clearly indicate that, in this study, PHO allelochemicals were highly disruptive to the community.

### Co-incubation with PHO Cells Does Not Lead to Loss in Diversity

When the community was inoculated with PHO cells (roughly 20% relative abundance right after inoculation) no marked effects were observed, and the PHO cells-exposed communities remained relatively stable in terms of composition (**Figure 3**), with the exception of a decrease in Actinobacteria and increase in Gammaproteobacteria that was nevertheless observed in the fresh medium extract treatment. Even though the relative abundance of PHO amplicons increased substantially between days 0 and 6, throughout the experiment the alpha diversity in the samples remained stable (**Figure 3A**). Moreover, despite this large abundance of PHO cells, beta diversity principal coordinates analysis (PCoA) indicates that on days 6 and 16 this treatment was considerably closer to the control (fresh medium) treatment, than the allelochemical-related treatments, although PCoA axis 2 indicates a gradient associated with the presence of PHO cells (**Figure 3C**). Hence, our observations dismiss a generalized allelopathic effect, comparable to the spent medium extract and portoamides A and B exposure treatments, for the co-incubation of the community with a considerable density of PHO cells. These observations contrast with previous observations in bialgal systems indicating that cell-to-cell contact may be required for allelopathic activity (Uchida et al., 1999; Dunker et al., 2017). Because PHO is able to produce allelochemicals as early as the third day of growth (Leão et al., 2010), it is unlikely that no effects were observed due to an inability of the cyanobacterium to produce the allelochemicals during the 16-day period of test. One possibility is that because the release of allelochemicals is likely slower than the one-time addition for the allelochemical-exposed treatments, degradation of allelochemicals by members of the microbial community (e.g., Christoffersen et al., 2002) prevents accumulation to toxic levels. Additionally, we speculate that the presence of certain organisms (and their infochemicals) or differences in nutrient availability (for example it is possible that, despite the small amount of fresh Z8 medium added, PHO cells could be nutrient limited toward the end of the exposure period) might have led to a downregulation of allelochemical biosynthesis. These latter hypotheses are perhaps counter-intuitive, as one would expect that cyanobacterial allelochemicals are produced precisely under stress conditions such as the presence of competitors, reduced light (shading) or nutrient limitation (Leflaive and Ten-Hage, 2007; Leão et al., 2009b) but will nevertheless be scrutinized in future work.

## Conclusion

Our findings indicate that compounds actively released by PHO under batch culture conditions are able to markedly influence the structuring of planktonic microbial communities. Members of the Cyanobacteria and Chlorophyta were strongly impacted by exposure, as expected from previous work, but certain heterotrophic bacteria groups were also very susceptible to allelochemicals released by PHO. The portoamides seem to be main drivers of these effects. However, in the presence of PHO cells and absence of exogenous allelochemicals, the microbial communities remained stable and similar to those exposed only to the fresh medium extract. It is difficult to anticipate how intricate the molecular- and organism-level processes behind this unexpected divergence are. Nevertheless, their study will likely reveal important clues regarding the role of allelopathy in shaping natural microbial planktonic communities.

## Author Contributions

FD: designed and conducted the experiments, analyzed data. JTA: designed and conducted experiments. TR and JA: carried out purification of the allelochemicals. VV: provided essential materials. PL: managed the project, designed experiments, analyzed data and led the writing of the manuscript. All authors contributed to the writing of the manuscript.

## Conflict of Interest Statement

The authors declare that the research was conducted in the absence of any commercial or financial relationships that could be construed as a potential conflict of interest.

## Footnotes

1 http://bbmap.sourceforge.net/
