# Supplementary material for: Cyanobacterial Allelochemicals But Not Cyanobacterial Cells Markedly Reduce Microbial Community Diversity
Source: Front Microbiol. 2017 Aug 8;8:1495. doi: 10.3389/fmicb.2017.01495 (PMC5550742; doi:10.3389/fmicb.2017.01495)
Supplement: Supplementary file 1 [file Image_1.PDF]

Supplementary Material

Cyanobacterial allelochemicals but not cyanobacterial cells markedly reduce microbial community diversity

Filipa Dias, Jorge T. Antunes, Tiago Ribeiro, Joana Azevedo, Vitor Vasconcelos, Pedro N. Leão*

*** Correspondence:**
Pedro N. Leão
pleao@ciimar.up.pt

1 Supplementary Figures

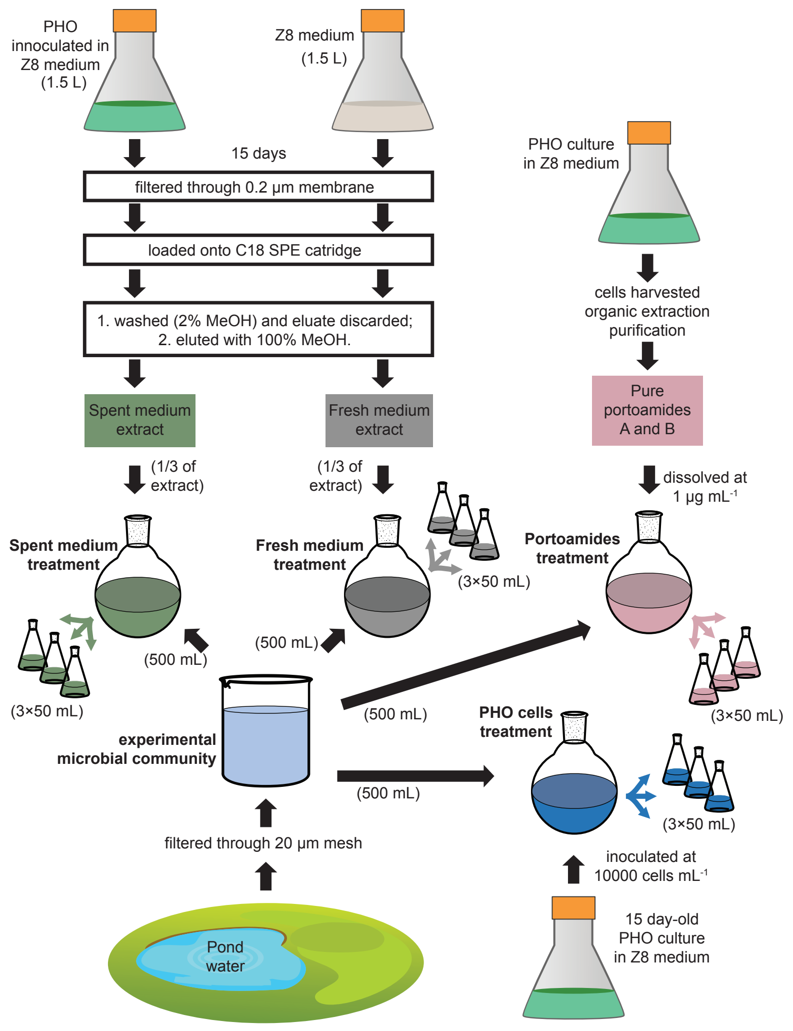

Supplementary Figure S1. Schematic representation of the experimental design for the exposure experiment.

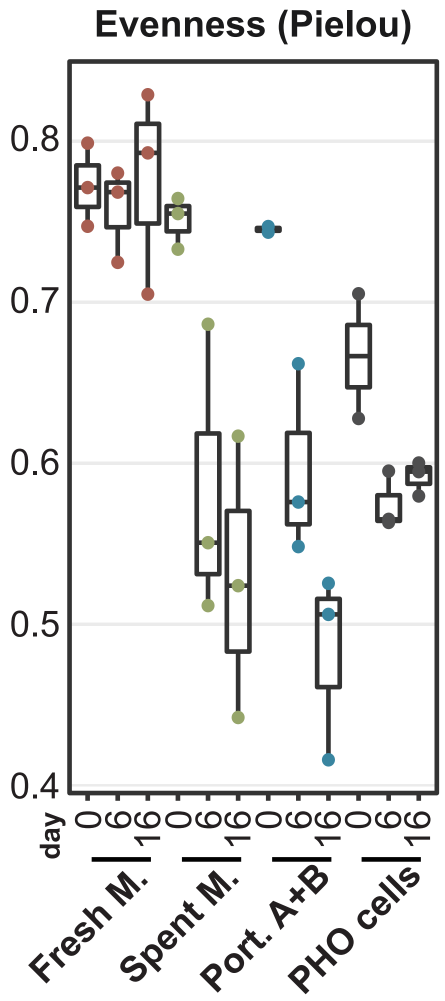

Supplementary Figure S2. Pielou evenness for the different treatments at different time points (calculated using the microbiome/microbiome package for R).
